# Tumour Progression Stage-Dependent Secretion of YB-1 Stimulates Melanoma Cell Migration and Invasion

**DOI:** 10.3390/cancers12082328

**Published:** 2020-08-18

**Authors:** Corinna Kosnopfel, Tobias Sinnberg, Birgit Sauer, Heike Niessner, Alina Muenchow, Birgit Fehrenbacher, Martin Schaller, Peter R. Mertens, Claus Garbe, Basant Kumar Thakur, Birgit Schittek

**Affiliations:** 1 Department of Dermatology, University of Tübingen, 72076 Tübingen, Germany; Tobias.Sinnberg@med.uni-tuebingen.de (T.S.); Birgit.Sauer@med.uni-tuebingen.de (B.S.); Heike.Niessner@med.uni-tuebingen.de (H.N.); alina.muenchow@student.uni-tuebingen.de (A.M.); Birgit.Fehrenbacher@med.uni-tuebingen.de (B.F.); Martin.Schaller@med.uni-tuebingen.de (M.S.); Claus.Garbe@med.uni-tuebingen.de (C.G.); 2 Department of Dermatology, University Hospital Würzburg, 97080 Würzburg, Germany; 3 Department of Nephrology and Hypertension, Diabetes and Endocrinology, Otto-von-Guericke University, 39120 Magdeburg, Germany; peter.mertens@med.ovgu.de; 4 Department of Pediatric Hematology and Oncology, University Hospital Essen, 45147 Essen, Germany; basant-kumar.thakur@uk-essen.de

**Keywords:** melanoma, secretion, Y-box binding protein 1, migration and invasiveness

## Abstract

Secreted factors play an important role in intercellular communication. Therefore, they are not only indispensable for the regulation of various physiological processes but can also decisively advance the development and progression of tumours. In the context of inflammatory disease, Y-box binding protein 1 (YB-1) is actively secreted and the extracellular protein promotes cell proliferation and migration. In malignant melanoma, intracellular YB-1 expression increases during melanoma progression and represents an unfavourable prognostic marker. Here, we show active secretion of YB-1 from melanoma cells as opposed to benign cells of the skin. Intriguingly, YB-1 secretion correlates with the stage of melanoma progression and depends on a calcium- and ATP-dependent non-classical secretory pathway leading to the occurrence of YB-1 in the extracellular space as a free protein. Along with an elevated YB-1 secretion of melanoma cells in the metastatic growth phase, extracellular YB-1 exerts a stimulating effect on melanoma cell migration, invasion, and tumourigenicity. Collectively, these data suggest that secreted YB-1 plays a functional role in melanoma cell biology, stimulating metastasis, and may serve as a novel biomarker in malignant melanoma that reflects tumour aggressiveness.

## 1. Introduction

The secretome of a cell represents the collective of all its factors released into the extracellular space. This includes extracellular matrix components, proteases, growth factors, cytokines, chemokines, as well as extracellular vesicles [1,2]. As an important pillar of cell–cell communication, these secreted factors can contribute to the acquisition and maintenance of the various cancer hallmarks defined by Hanahan and Weinberg in an autocrine, paracrine, or even endocrine manner [1,3,4,5]. This can happen by direct stimulation of tumour cell proliferation, survival, migration, and invasion. In addition, dynamic interaction between cancer cells and various cell types of the tumour stroma or at distant sites can facilitate a growth-promoting local tumour microenvironment, on the one hand, and the establishment of a pre-metastatic niche, on the other, supporting metastases formation [2,6].

In malignant melanoma, the tumour secretome has been shown to play an important role in the protection against targeted therapy with MAPK (mitogen-activated protein kinase) signalling pathway inhibitors [7,8,9,10]. Intriguingly, the adoption of pro-metastatic traits including mesenchymal properties and dedifferentiation of melanoma cells have been frequently described in therapy resistance [11,12,13]. Melanoma cell culture supernatants may induce a mesenchymal, tumour-initiating phenotype [14] and also directly stimulate tumour cell migration/invasion, neo-angiogenesis, and extracellular matrix remodelling. Consequently, melanoma cell culture supernatants can promote the metastatic potential of the cancer cells on several levels [14,15,16].

Previously, we could show that the oncogenic cold shock domain family member Y-box binding protein 1 (YB-1; Protein Accession: P67809) is overexpressed in malignant melanoma cells with further increasing protein levels during tumour progression [17,18]. Within the cell, YB-1 has been reported to control both translation processes in the cytoplasm and transcription in the nucleus throughout different cell types [19]. Accordingly, we found that in malignant melanoma, S102 phosphorylated nuclear YB-1 is associated with increased cell survival as well as therapy resistance towards chemotherapeutic agents and targeted therapies with MAPK pathway inhibitors [20,21], while unphosphorylated cytoplasmic YB-1 stimulates the migration, invasion, and tumourigenicity of melanoma cells [17]. In line with its versatile oncogenic function, elevated intracellular YB-1 represents an unfavourable prognostic marker in malignant melanoma [17].

In the context of inflammatory disease, YB-1 has been described to be actively secreted from various cell types promoting cell proliferation as well as migration and consequently propagating inflammatory processes [22,23]. Over the last years, there have been first reports of YB-1 species in the sera of patients suffering from advanced carcinomas and haematological malignancies [24,25,26]. Based on this, a possible extracellular occurrence of the cold shock domain protein in malignant melanoma as well as its potential oncogenic function is assessed in the present study.

## 2. Materials and Methods

### 2.1. Isolation and Culture of Human Cells

Melanoma cells were cultured in RPMI1640 (Gibco^TM^/Thermo Fisher Scientific; Waltham, MA, USA) supplemented with 10% fetal calf serum (Merck Biochrom; Berlin, Germany) and 1% penicillin/streptomycin (Gibco^TM^/Thermo Fisher Scientific; Waltham, MA, USA). Melanoma cell lines were purchased from ATCC (A375, SKMel28, SKMel19; Manassas, VA, USA), from DSMZ (MelJuso; Braunschweig, Germany), or kindly provided by the laboratory of M. Herlyn (Mel1617, 451LU, WM1346, WM266-4, 1205LU, WM115, WM793, WM1366, WM3211, WM1552, WM35, SbCl2; The Wistar Institute, PHL, USA). Cell cultures were regularly tested for mycoplasma using the Venor GeM Classic Mycoplasma Detection Kit (Minerva Biolabs; Berlin, Germany) and used no longer than two months upon thawing of the frozen stock.

Primary human melanocytes, fibroblasts, and keratinocytes were isolated and cultured as described earlier [20,27]. The use of human tissues was approved by the local medical ethical committee (16/2009B02) and experiments performed in accordance with the Declaration of Helsinki Principles.

### 2.2. Modulation of YB-1 Expression

Melanoma cells with inducible expression of YB-1 specific shRNA (shYB-1, TRIPZ, clone V2THS_232997) or non-silencing shRNA (NonSil, TRIPZ, #RHS4743 (Dharmacon/GE Healthcare; Lafayette, CO, USA)) were generated by lentiviral gene transfer and transgene expression induced by 2 µg/mL doxycycline (AppliChem; Darmstadt, Germany) in the culture medium as described earlier [17]. *YBX1* gene knockout (CRISPR *YBX1*^KO^) was conducted by CRISPR/Cas9 mediated genome engineering and single cell clones with effective *YBX1* knockout identified as described previously [21].

### 2.3. Lentiviral Gene Transfer

Lentiviral particles were produced in HEK293T cells (Biocat; Heidelberg, Germany) using a second-generation packaging system (psPAX2 and pCMV-VSV-G) and the respective lentiviral transfer vectors (TRIPZ-NonSil, TRIPZ-shYB-1, and lentiCRISPRv2-*YBX1* sgRNA1 and 2). Melanoma cells were transduced with lentivirus containing supernatants and, after three days, were selected for successful transduction with 2 µg/mL puromycin (InvivoGen; San Diego, CA, USA) in the cell culture medium.

### 2.4. YB-1 Secretion Assays

Conditioned cell culture supernatants for the assessment of YB-1 secretion were produced as follows: Cells of interest were seeded at 1.8 × 10^6^ cells (a) or 1 × 10^6^ cells (b) on 75 cm^2^ (a) or 25 cm^2^ cell culture flasks (b). After 16 h, cells were washed with PBS and serum-free cell culture medium was added—if applicable—containing the respective inhibitors or chemicals as indicated (brefeldin A (BD GolgiPlug^TM^), monensin (BD GolgiStop^TM^) (both BD Biosciences; Heidelberg, Germany); ionomycin, EGTA, MgCl_2_ (Sigma; Taufkirchen, Germany); BAPTA-AM (Invitrogen; Carlsbad, CA, USA)). Conditioned medium was removed at the respective time points (cell line panel: 48 h (a), secretion stimulation/inhibition: 4 h (b)) and cell debris removed by centrifugation at 1500× *g* for 10 min. Stimulation of cells with ATP (AppliChem; Darmstadt, Germany) was performed 30 min before supernatant collection.

For protease protection assays, supernatants were treated with either proteinase K (0.05 µg/µL; Sigma; Taufkirchen, Germany) for 10 min at 37 °C or trypsin (0.05%; Gibco^TM^/Thermo Fisher Scientific; Waltham, MA, USA) for 20 min at 37 °C. Protease activity was stopped by addition of PMSF (1 mM; Sigma; Taufkirchen, Germany) and incubation for 10 min at 95 °C. The lumenal exosomal protein TSG101 served as a positive control for intravesicular proteins and pre-treatment of supernatants with Triton X-100 (0.2%; AppliChem; Darmstadt, Germany) for 15 min on ice was conducted before protease digestion to confirm their effective degradation.

Concentration of the conditioned cell culture supernatants was performed by lyophilisation before analysis of YB-1 content by Western blot and ELISA.

### 2.5. Extracellular Vesicle Preparation/Clearance

Purification of extracellular vesicles (EVs) from conditioned cell culture supernatants was conducted using differential centrifugation followed by ultracentrifugation to collect exosomes containing EVs. Cells were removed by centrifuging at 500× *g* for 10 min at 10 °C. Cell debris was then removed by centrifuging the supernatant samples for 20 min at 3000× *g* at 10 °C. The supernatant was collected in new tubes and centrifuged at 12,000× *g* for 20 min at 10 °C to remove the apoptotic bodies and microvesicles. To obtain exosomes, supernatants were further ultracentrifuged using a fixed angle rotor (Beckman Coulter; Krefeld, Germany) at 100,000× *g* for 70 min at 10 °C. The resulting supernatants were collected as EV-depleted supernatants (cleared supernatant), while the EV pellets were washed by resuspension in 2 mL of PBS and ultracentrifugation of the samples at 100,000× *g* for 70 min at 10 °C. After discarding the supernatant, the final exosome containing EV pellet was resuspended in 200 μL of PBS for downstream analysis. Equal volumes of unfractionated (total) and cleared supernatants as well as equivalent volumes of purified EVs were assessed for YB-1 content by Western blot analysis. The lumenal exosomal marker TSG101 served as a control for successful purification or depletion of vesicles.

### 2.6. Western Blotting

Lyophilised cell culture supernatants were resuspended in 2 × Lämmli Buffer (0.1 M Tris base pH6.8; 4% (*w*/*v*) SDS, 10% (*v*/*v*) glycerol, 10% (*v*/*v*) β-mercaptoethanol, 0.5 mg/mL bromophenol blue), and equivalents of 250 µL supernatant subjected to SDS-PAGE with subsequent transfer of proteins onto a PVDF membrane (Roche; Basel, Switzerland). After blocking with 5% dry milk in PBS-T (0.1% Tween-20), the following primary antibodies were applied: anti-YB-1 (C-terminal (CT), ab76149, Abcam, Cambridge, UK; N-terminal (NT), Eurogentec, Liège, Belgium), anti-GAPDH (#2118, Cell Signaling Technology, Leiden, The Netherlands), anti-Lamin B (sc6216), and anti-TSG101 (sc7964) (both Santa Cruz Biotechnology; Heidelberg, Germany). Immunodetection was conducted using anti-rabbit, anti-mouse (Cell Signaling Technology; Leiden, The Netherlands), or anti-goat (Santa Cruz Biotechnology; Heidelberg, Germany) IgG horse radish peroxidase (HRP)-conjugated secondary antibodies and Pierce^TM^ ECL Western Blotting Substrate or the SuperSignal^TM^ West Dura Extended Duration Substrate (both Thermo Fisher Scientific; Waltham, MA, USA). Immunoreactive bands were densitometrically assessed using ImageJ (Wayne Rasband, NIH; Bethesda, MD, USA) and YB-1 content was calculated based on a recombinant GST-tagged YB-1 protein control (Abnova; Heidelberg, Germany). Cells and extracellular vesicle fractions were lysed with RIPA lysis buffer (25 mM Tris HCl pH 7.6, 150 mM NaCl, 1% NP-40, 1% sodium deoxycholate, 0.1% SDS) containing protease (cOmplete, Roche; Basel, Switzerland) and phosphatase inhibitors (PhosSTOP, Roche; Basel, Switzerland) before supplementation with Lämmli Buffer and subsequent assessment in Western blot.

To assess the total protein content of the conditioned culture supernatants, membranes were stained with Coomassie Brilliant Blue R-250 staining solution (Bio-Rad; Feldkirchen, Germany) before blocking as described in Goldman et al. [28].

### 2.7. ELISA

Lyophilised cell culture supernatants were resuspended in water and equivalents of 125 µL of supernatant were analysed for YB-1 content. To this end, 96-well plate cavities were coated with either supernatant samples or recombinant YB-1 standard (0–250 ng/mL, Abnova) (16 h, + 4 °C), blocked (1 h; 2% BSA/PBS), incubated with YB-1 specific antibody (ab76149, Abcam; Cambridge, UK) in 0.5% BSA/0.05% Tween-20/PBS (1 h) and with HRP-conjugated secondary antibody (Cell Signaling Technology; Leiden, The Netherlands) in 0.5% BSA/0.05% Tween-20/PBS (1 h). The incubation with detection solution (TMB, Cell Signaling Technology; Leiden, The Netherlands) was stopped with 1 M sulphuric acid and absorption at 450 nm was measured with a TriStar LB941 microplate reader (Berthold Technologies; Bad Wildbad, Germany).

### 2.8. Electron Microscopy

Cells were stimulated with ionomycin (2 h) or ATP (15 min) in serum-free cell culture medium. For pre-embedding immunogold labelling, cells were fixed in 4% paraformaldehyde (30 min), permeabilised with 0.2% Triton X-100/PBS (10 min, on ice), incubated with YB-1 specific antibody (ab76149, Abcam; Cambridge, UK) in PBS (30 min), and stained with secondary antibody conjugated to 1 nm colloidal gold particles (Nanoprobes; Yaphank, NY, USA) (30 min). After fixation with 2.5% glutaraldehyde (90 min) and silver enhancement with HQ silver enhancement kit (Nanoprobes; Yaphank, NY, USA), cells were fixed again in Karnovsky’s fixative and stored at 4 °C. Cell pellets were embedded in 3.5% agarose at 37 °C, coagulated at room temperature, and fixed again in Karnovsky’s solution. Post-fixed samples (1% OsO_4_, 20 min, on ice) were rinsed with distilled water, block-stained with uranyl acetate (2% in distilled water), and dehydrated in ascending series of alcohol dilutions to 100%. Samples were infiltrated with propylene oxide, embedded in glycide ether, and cut using an ultramicrotome (Ultracut; Reichert-Jung/Leica Microsystems; Wetzlar, Germany). Ultrathin sections (30 nm) were mounted on copper grids and analysed using a Zeiss LIBRA 120 transmission electron microscope (Carl Zeiss; Oberkochen, Germany) operating at 120 kV.

### 2.9. Recombinant Protein Preparation

Recombinant FLAG-tagged YB-1 was produced in HEK293T cells using the pDream2.1/YB-1 expression vector (based on GenScript pDream 2.1, kindly provided by P.R. Mertens). Cells were lysed in 50 mM Tris-HCl pH 7.4, 1 mM EDTA, 150 mM NaCl, 1% Triton X-100 in water supplemented with protease inhibitors (cOmplete, Roche; Basel, Switzerland). Recombinant YB-1 was purified by binding to anti-FLAG M2 affinity gel (Sigma; Taufkirchen, Germany) with subsequent FLAG-peptide mediated (100 µg/mL; ApexBio; Houston, TX, USA) competitive elution in TBS containing protease inhibitors. The eluate was concentrated, and FLAG peptide was depleted by filter centrifugation with Vivaspin 6 Filterfalcons (10 kda cut-off; Sartorius; Göttingen, Germany). Protein concentration was determined with the Pierce^TM^ BCA Protein Assay Kit (Thermo Fisher Scientific; Waltham, MA, USA).

In the functional assessment of rYB-1, equivalent concentrations of Albumin Fraction V (BSA, Roth; Karlsruhe, Germany) dissolved in TBS were used as a control for unspecific effects attributed to protein supplementation. To further confirm specificity of the observed effects, recombinant YB-1 was heat denatured or concomitantly applied with a YB-1 specific blocking antibody (sc-398340, Santa Cruz Biotechnology; Heidelberg, Germany) or its matched isotype control (mouse IgG2a κ light chain antibody).

### 2.10. Viability Assays

Cell viability was routinely assessed after collection of the conditioned cell culture supernatants by visual analysis and staining of cells with compromised membrane integrity using trypan blue (1:10 dilution of 0.4% solution; Sigma; Taufkirchen, Germany) with subsequent microscopic quantification and/or flow cytometric assessment following propidium iodide staining (1 µg/mL in PBS, 5 min; Sigma; Taufkirchen, Germany).

Cell culture supernatants were analysed for LDH release as a correlate for cell death with the LDH Cytotoxicity Detection Kit (Roche). LDH release was determined relative to a 1% Triton X-100 (AppliChem; Darmstadt, Germany) pre-treated death control.

Intracellular ATP content was quantified with the CellTiterGlo Luminescent Cell Viability Assay (Promega; Walldorf, Germany).

To assess the effect of extracellular recombinant YB-1 (rYB-1) or downregulation of intracellular YB-1 on the number of viable cells over time, 1.5 × 10^3^ cells were seeded in 96 well plate cavities. If applicable, cells were treated with rYB-1, BSA, or 10% FCS in serum-free culture medium 16 h after seeding. Cell viability was assessed at the indicated timepoints after treatment or seeding by washing twice with PBS and incubation with 100 µL of 100 µg/mL 4-methylumbelliferyl-hepanoate (MUH) in PBS for 1 h at 37 °C. Fluorescence (λ_ex_ 355 nm, λ_em_ 460 nm) as a correlate for the number of viable cells was measured with a TriStar LB941 fluorescence microplate reader (Berthold Technologies; Bad Wildbad, Germany).

### 2.11. Cell Cycle Analysis

2.5 × 10^5^ cells were seeded in 6-well cavities. If applicable, after 16 h, the indicated treatments were applied in serum-free medium. Cells were harvested and permeabilised with 70% ice cold ethanol. After staining with 50 µg/mL propidium iodide (Sigma; Taufkirchen, Germany) and 100 µg/mL RNAse A (AppliChem; Darmstadt, Germany) in PBS for 30 min, the distribution of the cells in the different cell cycle phases was detected with a BD^TM^LSR II flow cytometer and FACSDiva^TM^ software (both BD Biosciences; Heidelberg, Germany).

### 2.12. Wound Healing Assay

Melanoma cell migration into an artificially introduced scratch within a confluent cell layer was assessed as described before [17]. Relative wound closure over time was evaluated with ImageJ (Wayne Rasband, NIH; Bethesda, MD, USA).

### 2.13. Migration and Matrigel Invasion Assays

Boyden chamber-based migration and Matrigel invasion assays were performed as described previously [17] using 1 × 10^5^ MelJuso, 5 × 10^4^ WM266-4 or 3.5 × 10^4^ WM1366 melanoma cells per transwell insert.

### 2.14. Anchorage-Independent Growth Assay

2.5 × 10^3^ melanoma cells were seeded in 500 µL of 0.5% agar noble (BD Difco/Thermo Fisher Scientific; Waltham, MA, USA) in serum-free medium into cavities of a 12-well plate pre-coated with 750 µL 0.5% agar noble. After 6 h, 750 µL of culture medium was added with the indicated treatments and exchanged twice a week. After 10 d, cells were fixed and stained with 0.0023% Crystal Violet (Sigma-Aldrich; Taufkirchen, Germany) in 4% paraformaldehyde. The colonies formed in each well were counted with a phase-contrast CK40 culture microscope (Olympus; Hamburg, Germany).

### 2.15. Statistical Analysis

Statistical analysis was conducted with GraphPad Prism version 8.4 (GraphPad Software). Where applicable, one-way ANOVA with subsequent Tukey’s multiple comparisons tests was used for *p*-value calculation and significance determination. *p*-values < 0.05 were considered statistically significant (* for *p* < 0.05, ** for *p* < 0.01, *** for *p* < 0.001, **** for *p* < 0.0001).

## 3. Results

### 3.1. YB-1 Is Secreted by Melanoma Cells in a Progression Stage-Dependent Manner

**Figure 1:**
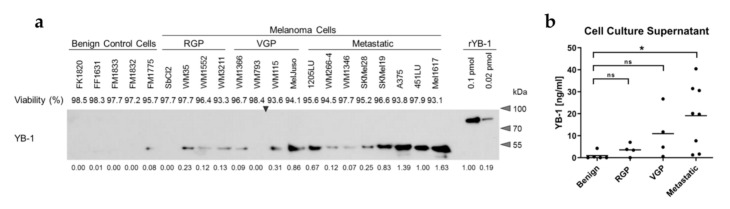
YB-1 is secreted by melanoma cells in a progression stage-dependent manner. (**a**,**b**) Immunoblot analysis of 48 h-conditioned serum-free cell culture supernatants of benign control cells (melanocytes (FM), fibroblasts (FF), keratinocytes (FK)) and of melanoma cell lines derived from metastatic melanomas or primary melanomas in the radial (RGP) or vertical growth phase (VGP). A representative immunoblot using an antibody targeting the C-terminus of YB-1 is shown (**a**, uncropped Western blot in Figure S1a). An arrowhead marks the boundary between the two in parallel processed gels. Densitometric analysis was conducted on four independent experiments (relative values of the prevalent experiment indicated below the immunoblot). YB-1 concentrations in the supernatants was approximated with the help of a recombinant YB-1 protein standard included on each gel (**b**; mean values for individual cell lines (points) and group means (vertical lines) are shown). Significance was determined with one-way ANOVA and subsequent Tukey’s multiple comparison test (ns = non-significant, * for *p* < 0.05, ** for *p* < 0.01, *** for *p* < 0.001 and **** for *p* < 0.0001). Relative viability was assessed by LDH release and trypan blue uptake (**a**).

To evaluate a potential secretion of YB-1 from melanoma cells, conditioned serum-free cell culture supernatants were generated using a panel of melanoma cell lines as well as melanocytes (FM), keratinocytes (FK), and fibroblasts (FF) as benign control cells of the skin. While YB-1 was readily detectable in the culture supernatants of many melanoma cell lines using both Western blot analysis (Figure 1a,b; Figure S1a,b) as well as ELISA (Figure S2a), this was generally not the case for the benign control cells. Interestingly, the amount of secreted YB-1 seems to increase with melanoma progression from radial growth phase (RGP) over vertical growth phase (VGP) to the metastatic stage. Accordingly, YB-1 was barely detectable in supernatants of WM793 cells, whereas 1205LU melanoma cells, which were derived from this primary melanoma cell line through passaging in mice and selection for lung metastasis [29], exhibited robust YB-1 secretion. Of note, the differences in extracellular YB-1 occurrence were not related to differences in the viability of the cells used for the generation of the conditioned supernatants as investigated by assessing the integrity of the cell membranes via quantification of lactate dehydrogenase (LDH) release and cellular trypan blue uptake (Figure 1a). This was further confirmed by the absence of Lamin B in the supernatants (Figure S1b). As a nuclear envelope protein, Lamin B serves as a marker for non-specific protein release into the extracellular space, while being easily detectable in cellular lysates. A comparison of culture supernatant conditioned by WM1366 melanoma cells with unconditioned medium supplemented with WM1366 cell lysate mimicking 10% of cell death accordingly shows markedly enhanced Lamin B occurrence upon the loss of cellular integrity by cell lysis (Figure S2b). At the same time, YB-1 levels in the conditioned supernatant were comparable to the 10% cell lysis control, indicating additional mechanisms beyond passive protein release leading to the presence of extracellular YB-1 in the melanoma cell samples. Similarly, the enhanced YB-1 release during melanoma progression cannot be explained by a generally elevated protein secretion from the tumour cells as assessed by Coomassie staining of the size-separated supernatant samples (Figure S2c).

A cross-detection of structurally related cold-shock proteins such as Y-box binding protein 3 (DbpA, P16989) in the melanoma cell culture supernatants can further be excluded, since Western blot analysis was performed with both antibodies targeting the C-terminus and the non-conserved N-terminus of YB-1 (Protein Accession: P67809) (Figure S1b). Overall, this indicates a specific secretion of YB-1 from melanoma cells increasing with tumour progression.

### 3.2. YB-1 Secretion Is Independent of the Classical ER/Golgi Mediated Secretory Pathway and Stimulated by Elevated Intracellular Ca^2+^ and ATP

**Figure 2:**
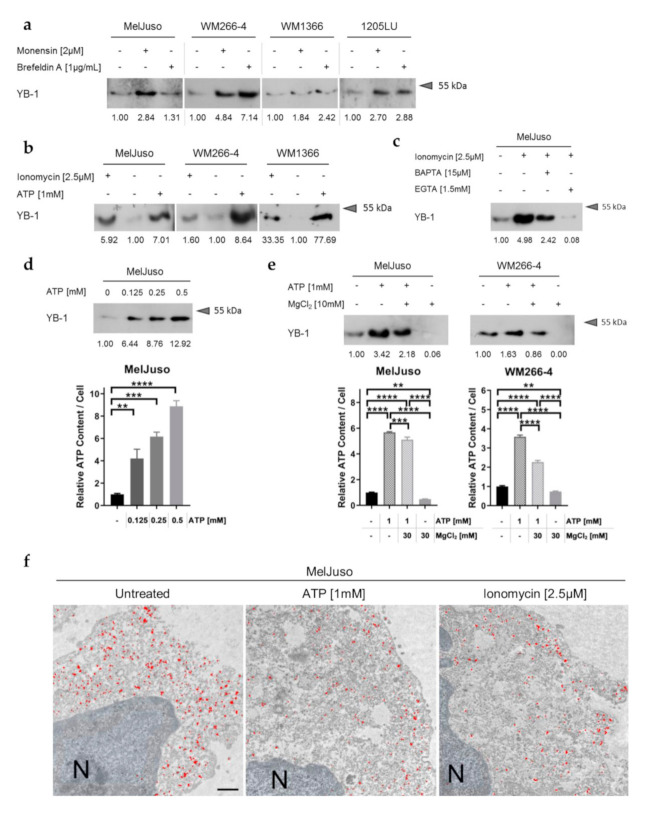
YB-1 secretion is mediated by a calcium- and ATP-dependent non-classical secretory pathway. (**a**) Western blot analysis of serum-free cell culture supernatants after inhibition of classical protein secretion by monensin or brefeldin A. Representative data of at least two independent experiments are shown. Relative band intensities are indicated relative to the untreated control supernatants and uncropped Western blots available in Figure S3a. (**b**–**e**) Western blot analysis of serum-free culture supernatants after stimulation of the melanoma cells with the Ca^2+^ ionophore ionomycin and ATP (**b**), the combination of ionomycin and the calcium chelators BAPTA or EGTA (**c**), with increasing ATP concentrations (**d**), or the combination of ATP and the ATP complexing agent MgCl_2_ (**e**). Relative band intensities are indicated and uncropped immunoblots presented in Figure S4. The intracellular ATP content was measured with the CellTiter-Glo^®^ Luminescent Cell Viability Assay (Promega) (**d**,**e**, bottom; mean ± standard deviation (SD), representative experiment of N = 2 with *n* = 3). Significance was determined with one-way ANOVA and subsequent Tukey’s multiple comparison test (ns = non-significant, * for *p* < 0.05, ** for *p* < 0.01, *** for *p* < 0.001 and **** for *p* < 0.0001). (**f**) Electron micrographs of a YB-1 specific immunogold labelling (C-terminal-antibody; red) in MelJuso cells stimulated with either ATP or ionomycin. Nucleus (N), scale bars represent 0.5 µm.

In pursuit of the mechanism underlying active YB-1 release from melanoma cells, first, a possible involvement of the classical pathway of secretion was assessed. However, inhibition of this classical secretory route via endoplasmic reticulum (ER) and Golgi apparatus using brefeldin A and monensin could not prevent or impair YB-1 release from different melanoma cell lines (Figure 2a; Figure S3a). On the contrary, even increased extracellular YB-1 could be detected in some cases, which did not correlate with cell death induction upon inhibition of classical protein secretion (Figure S3b). Similar observations have been made for proteins secreted via unconventional pathways (non-classical protein secretion) such as interleukin-1β (IL-1β) and macrophage migration inhibitory factor (MIF) [30,31]. Accordingly, YB-1 lacks a signal peptide for classical protein secretion as shown by in silico analysis using SignalP5.0 (likelihood of signal peptide: 0.0024; Figure S3c) [32].

At the same time, the bioinformatic tool SecretomeP 2.0 predicts non-classical secretion of YB-1 (NN-score: 0.733, above threshold of 0.6; Figure S3d) [33], in line with previous observations in an inflammatory context [22]. Similarly, secretion of YB-1 from melanoma cells could be promoted by increased intracellular calcium (Ca^2+^) and ATP levels as shown by stimulation with the calcium ionophore ionomycin as well as with ATP throughout different cell lines (Figure 2b; Figure S4a,b). Concomitant treatment with the Ca^2+^ chelators BAPTA and EGTA diminished the stimulating effect of ionomycin, corroborating the importance of freely available intracellular calcium in YB-1 secretion from melanoma cells (Figure 2c; Figure S4c,d). Analogously, the dose-dependent effect of ATP on YB-1 release from the cells (Figure 2d; Figure S4e) was attenuated by treatment with MgCl_2_ as an ATP complexing agent (Figure 2e; Figure S4d). Corresponding to a correlation of intracellular ATP levels and the amount of extracellular YB-1 (Figure 2d,e; bottom), MgCl_2_ treatment was also capable of reducing basal YB-1 secretion (Figure 2e; Figure S4d). Electron microscopy of intracellular YB-1 distribution further reveals mobilisation of YB-1 to the cytoplasmatic membrane upon stimulation with ionomycin or ATP, which may serve as a correlate for ongoing secretion (Figure 2f).

### 3.3. Secreted YB-1 Occurs Mainly as a Free Protein

**Figure 3:**
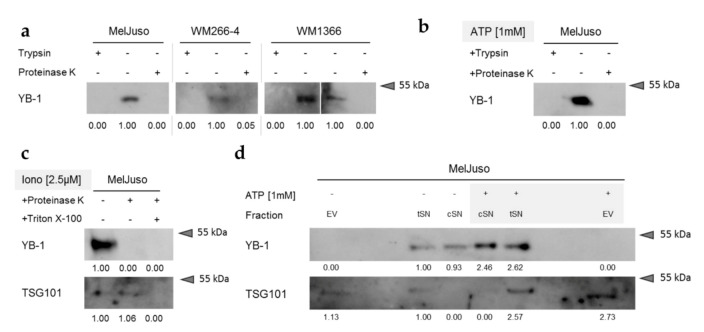
YB-1 secreted from melanoma cells occurs mainly as a free protein. (**a**–**c**) Protease protection assay followed by Western blot analysis for YB-1 in serum-free cell culture supernatants. The cells remained untreated (**a**), were stimulated with ATP [1 mM] (**b**) or were stimulated with ionomycin [2.5 µM] (**c**). Culture supernatants were proteinase K (**a**–**c**) or trypsin (**a**,**b**) digested in the absence or presence of Triton X-100 (**c**) to disrupt vesicular membranes. The lumenal exosomal marker TSG101 serves as a positive control for intravesicular proteins. Relative band intensities are indicated relative to the untreated control supernatants and uncropped Western blots available in Figure S5. (**d**) Western blot analysis of MelJuso-conditioned serum-free cell culture supernatants before (total SN, tSN) and after (cleared SN, cSN) purification of extracellular vesicles (EV) by ultracentrifugation. The lumenal exosomal marker TSG101 serves as control for the successful purification or depletion of vesicles. Relative band intensities are indicated relative to the tSN of untreated cells and uncropped immunoblots available in Figure S6. Representative data of two independent experiments are shown.

To further characterise the underlying mechanism of YB-1 secretion from melanoma cells, we next assessed the form of its extracellular presence—that is, its potential packaging into vesicles. As shown by protease protection assays using proteinase K or trypsin for digestion, YB-1 in melanoma cell culture supernatants is readily degraded, suggesting its extracellular occurrence as a free protein (Figure 3a; Figure S5a). This was not only the case for basal YB-1 secretion, but also after stimulation of YB-1 release by ATP (Figure 3b; Figure S5a) and ionomycin (Figure 3c; Figure S5b). The validity of the protease protection assay was confirmed using the exosomal lumenal marker protein TSG101, which is protected from protease digestion in the conditioned cell culture supernatant but can be targeted for degradation after disruption of vesicular membranes by Triton X-100 treatment (Figure 3c; Figure S5b).

In agreement with this, clearance of extracellular vesicles (EVs) from the conditioned supernatants (total supernatants, tSN → cleared supernatants, cSN) did not affect their YB-1 content, neither the basal levels, nor those after stimulation of YB-1 secretion with ATP (Figure 3d; Figure S6). Accordingly, YB-1 could not be detected in equivalent volumes of the corresponding purified EV fractions. Presence of the exosomal marker TSG101 in the EV fraction as well as its absence from the cSN samples corroborated the successful vesicle purification / depletion. The consistent results of protease protection assay and EV purification strongly suggest that YB-1 predominantly occurs as a free protein in melanoma cell culture supernatants with similar mechanisms at work in both basal and stimulated YB-1 secretion.

### 3.4. Extracellular YB-1 Stimulates Melanoma Cell Migration

**Figure 4:**
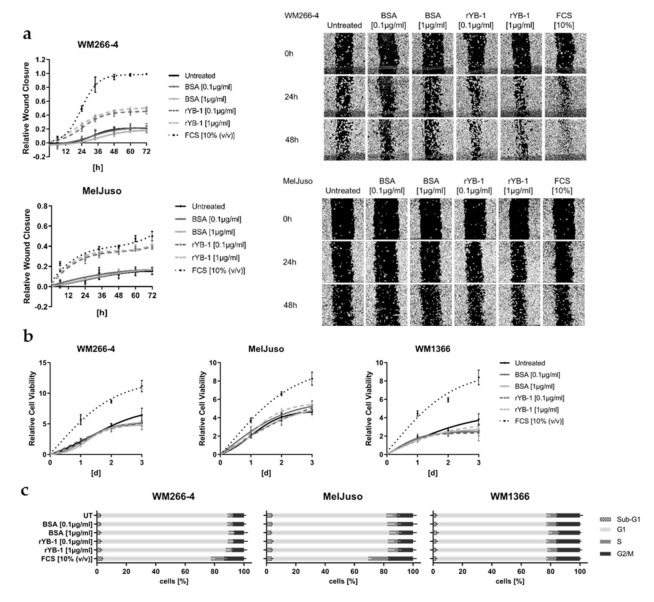
Extracellular YB-1 stimulates wound healing capacity but not proliferation of melanoma cells. (**a**) Analysis of melanoma cell mediated wound closure after stimulation with recombinant YB-1 (0.1 µg/mL, 1 µg/mL), BSA as protein control (0.1 µg/mL, 1 µg/mL), or 10% FCS as positive control compared to the untreated (UT) cells (mean ± SD; N = 2 with *n* = 8; left panel). Representative pictures are shown after 0, 24, and 48 h (right panel). (**b**) Cell viability-based growth curves after stimulation with rYB-1 (0.1 µg/mL, 1 µg/mL), BSA (0.1 µg/mL, 1 µg/mL), or 10% FCS over 3 d. Representative data of two independent experiments are shown (mean ± SD, *n* = 6). (**c**) Flow cytometric cell cycle analysis of melanoma cells stimulated with rYB-1 (0.1 µg/mL, 1 µg/mL), BSA (0.1 µg/mL, 1 µg/mL), or 10% FCS for 24 h. Fractions of cells in sub-G1, G1, S, and G2/M phase were quantified (mean ± SD, N = 2 with *n* = 3).

Based on the increasing amounts of YB-1 secreted from melanoma cell lines derived from invasively growing primary (VGP) and metastatic tumours, a potential effect of extracellular YB-1 on melanoma cell migration was assessed. Using both a VGP (MelJuso) as well as a metastatic melanoma cell line (WM266-4), we can show that extracellular stimulation of melanoma cells with recombinant YB-1 (rYB-1) accelerates closure of an artificially introduced wound in the cell layer (Figure 4a). This could not be observed after stimulation with bovine serum albumin (BSA) as a control for unspecific protein stimulation. Treatment with rYB-1 did not affect melanoma cell proliferation, as assessed by viability-based growth curves and cell cycle analyses (Figure 4b,c), indicating that the observed effect on wound closure can be attributed to an enhanced migration of the melanoma cells into the wound.

**Figure 5:**
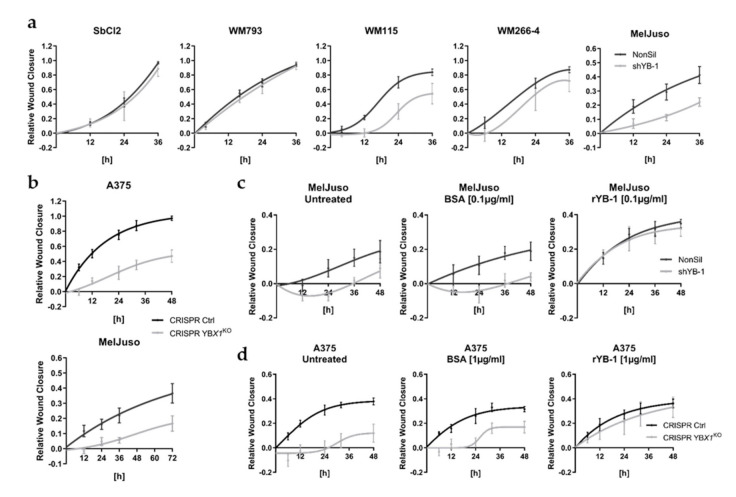
Melanoma cell migration is stimulated by extracellular YB-1. (**a**,**b**) Wound closure by different melanoma cell lines with downregulated YB-1 expression compared to the respective control cells (mean ± SD). shYB-1-based RNA interference (**a**; *n* = 6 (SbCl2, WM115) or *n* = 8 (WM793, WM266-4, MelJuso)), CRISPR/Cas9 mediated *YBX1* knockout (**b**; *n* = 4 (A375), *n* = 8 (MelJuso)). (**c**,**d**) Comparison of melanoma cells with shRNA-induced downregulation of YB-1 (shYB-1) and control shRNA-expressing cells (NonSil) (**c**) or with CRISPR/Cas9 mediated *YBX1*^KO^ and the corresponding control cells (**d**) under the respective treatment conditions (mean ± SD; *n* = 8).

Previous studies have already revealed a crucial role of YB-1 within metastatic melanoma cells associated with their migratory and invasive capacity [17,20]. Accordingly, upon downregulation of intracellular YB-1 levels by means of RNA interference (Figure S7) or CRISPR/Cas9 mediated *YBX1* knockout (Figure S8), we found an impaired wound closure capacity for several melanoma cell lines (A375, MelJuso, WM115, WM266-4) (Figure 5a,b; Figure S9a,b). In line with earlier reports, this was not associated with differences in melanoma cell growth by manipulation of YB-1 expression (Figure S10a,b) [17,21]. As suggested by the absent impact of YB-1 knockdown on wound closure in melanoma cell lines lacking a substantial YB-1 secretion (SbCl2, WM793; Figure 1, Figure 5a; Figure S9a), the presence of an effect correlates with the intrinsic secretory capacity of YB-1 in the different melanoma cells (SbCl2, WM793 < WM266-4 < WM115, MelJuso, A375), which in turn hints to an involvement of extracellularly occurring YB-1. Correspondingly, we can show here not only that decreased amounts of intracellular YB-1 go along with its reduced extracellular occurrence (Figure S10c,d), but also that the melanoma cell lines used in the above mentioned previous studies (A375, 1205LU, SKMel28) [17,20] exhibit a robust intrinsic YB-1 secretion (Figure 1).

Vice versa, addition of recombinant YB-1 (rYB-1) to the culture medium could rescue the decreased migratory capacity upon downregulation of YB-1 in melanoma cell lines with robust YB-1 secretion (Figure 5c,d; Figure S9c). Again, rYB-1 stimulation had no effect on proliferation of the melanoma cells, irrespective of their intracellular YB-1 content (Figure S11a,b). While rYB-1 already promoted wound closure at concentrations as low as 10 ng/mL, its pro-migratory impact could be reversed by both heat denaturation of rYB-1 and YB-1 specific blocking antibodies (Figure S12a–c), corroborating the specificity of the observed effect.

### 3.5. Recombinant YB-1 Promotes the Metastatic Capacity of Melanoma Cells

**Figure 6:**
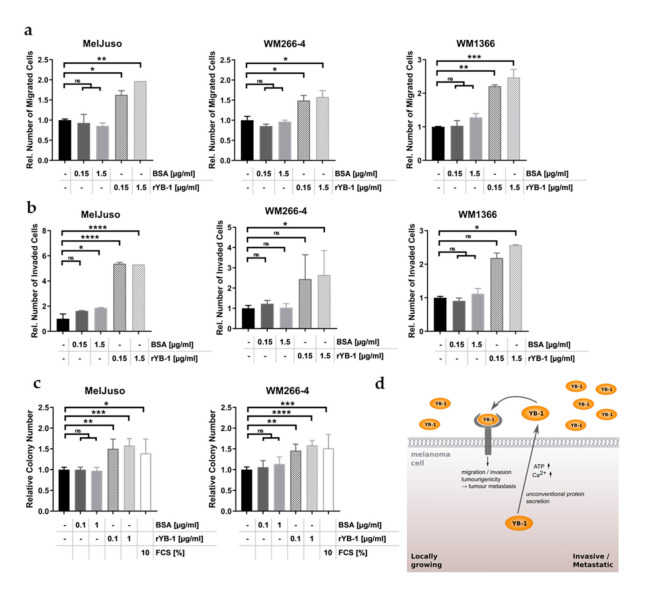
Extracellular YB-1 stimulates migration, invasion, and in vitro tumourigenicity of melanoma cells. (**a**,**b**) Boyden chamber-based cell migration (**a**) or invasion assay (**b**) after stimulation with rYB-1 (0.1 µg/mL, 1 µg/mL) or BSA (0.1 µg/mL, 1 µg/mL). The relative number of migrated (**a**) and invaded (**b**) cells was calculated based on the evaluation of five optical fields and normalised to the respective untreated controls (mean ± SD, *n* = 2). Significance was determined by one-way ANOVA with subsequent Tukey’s multiple comparisons test (ns = non-significant, * for *p* < 0.05, ** for *p* < 0.01, *** for *p* < 0.001 and **** for *p* < 0.0001). (**c**) Anchorage-independent growth assays of melanoma cells stimulated with rYB-1 (0.1 µg/mL, 1 µg/mL), BSA (0.1 µg/mL, 1 µg/mL), or 10% FCS. Colony numbers were normalised to the respective untreated controls (mean ± SD, N = 2 with *n* = 2). One-way ANOVA with Tukey’s multiple comparisons test was used to assess significant differences (ns = non-significant, * for *p* < 0.05, ** for *p* < 0.01, *** for *p* < 0.001 and **** for *p* < 0.0001). (**d**) Schematic graph depicting the functional effects of extracellular YB-1 as well as its secretion from melanoma cells.

As extracellular YB-1 did not alter melanoma cell growth (Figure 4b,c; Figure S11a,b), its accelerating effect on wound closure presumably relies on an enhanced migratory capacity of the cells. Accordingly, stimulation with recombinant YB-1 significantly enhanced the number of migrated melanoma cells in Boyden chamber-based migration assays (Figure 6a). This applied both to melanoma cell lines with robust YB-1 secretion (MelJuso) and to those which intrinsically release comparatively small amounts of YB-1 into their supernatant (WM266-4, WM1366). Intriguingly, the stimulatory effect of recombinant YB-1 could be extended not only to the invasiveness of the melanoma cells (Figure 6b), but also to their in vitro tumourigenicity, i.e., their capacity to grow in an anchorage-independent manner (Figure 6c). Based on this and in connection with the marked increase in YB-1 secretion during tumour progression, extracellular YB-1 appears to play an essential role in promoting the metastatic process of melanoma cells (Figure 6d).

## 4. Discussion

The Y-box binding protein 1 is a multifunctional protein whose expression is significantly increased in a great number of tumour entities [19]. In line with its frequent association with poor prognosis and disease recurrence, intracellular YB-1 has been described to promote the metastatic capacity in various cancer systems [19]. This includes, on the one hand, an enforced activity of matrix metalloproteinases by YB-1 dependent transcriptional induction and altered intracellular trafficking [34,35]. On the other hand, YB-1 is capable of inducing a highly motile and invasive mesenchymal phenotype in epithelial tumours such as breast, prostate, and gastric carcinomas, as well as in malignant melanoma and sarcoma [17,36,37,38,39]. In this context, cytoplasmic YB-1 has been shown to promote the translation of mRNAs encoding both epithelial-to-mesenchymal transition (EMT)-regulating (e.g., Snail, Twist, Zeb2/Sip1) and cytoprotective factors facilitating tumour cell metastasis [37,39,40,41,42].

First evidence for an additional extracellular role of YB-1 was provided in an inflammatory context with active secretion of the cold-shock domain protein from various cell types including granulocytes, lymphocytes, mesangial, liver, and monocytic cells following endotoxin exposure [22,23]. In agreement with its RNA binding capacity, YB-1 has been further shown to be a component of exosomes involved in sorting and packaging of mRNAs and miRNAs [43,44]. Although YB-1 species have been reported to occur in sera from tumour patients suffering from advanced carcinomas and haematological malignancies [24,25,26], the underlying cells of origin and secretion mechanisms in this context remain largely unexplored. Here, we can show an active secretion of full-length YB-1 from melanoma cells. Similar to the secretion of YB-1 from inflammatory cells and in line with the absence of a signal peptide at its N-terminus, this was mediated by unconventional protein secretion [22].

The process of unconventional protein secretion is complex (reviewed in Nickel and Rabouille [45]): The release of leaderless proteins into the extracellular space as free proteins can be sub-classified into three distinct mechanisms involving translocation across the plasma membranes via pores (type I) or ABC transporters (type II) as well as the translocation across the membrane of intracellular vesicles, which subsequently merge with the plasma membrane (type III). To further complicate matters, leaderless proteins can also exit the cells packed into extracellular vesicles (EVs), including exosomes and microvesicles. Despite recent progress, the exact mechanisms underlying unconventional secretion of many proteins still remain elusive and can even differ between different cell types as it is the case for interleukin-1β [46,47]. This also seems to be the case for YB-1. While its secretion is stimulated by increased intracellular ATP and Ca^2+^-levels in both inflammatory and melanoma cells, YB-1 is released as a free protein into the extracellular space of the latter as opposed to its intravesicular occurrence in the monocyte supernatant [22]. Altogether, the mechanism of YB-1 release from melanoma cells shows similarities to the process of FGF2 secretion, which is ATP-dependent and involves its recruitment to the plasma membrane [48,49]. FGF2 has been further shown to interact with phosphatidylinositol-4,5-bisphosphonate [PI(4,5)P_2_] in the plasma membrane and to exploit self-oligomerisation mediated pore formation to exit the cells [48,50,51]. Intriguingly, YB-1 does not only exhibit a high tendency for multimerisation but also possesses positively charged, polybasic clusters in its C-terminus, which could serve as PI(4,5)P_2_ binding domains [19,52,53]. Yet, since neither lipid binding nor pore formation by YB-1 has been determined to date, the exact mode of its secretion in tumour cells should be the subject of future studies.

Whereas extracellular YB-1 exerted a pro-proliferative function in an inflammatory setting and inhibited proliferation of receiving cells following its secretion in response to oxidative insults [22,54], we could not detect such effects on melanoma cell growth. This is in agreement with the herein presented and our previous data reporting constant melanoma cell proliferation upon modulation of intracellular YB-1 levels, which also influences the amount of extracellular YB-1 [17,21]. Earlier, we could show that increased YB-1 expression of melanoma cells stimulates their therapy resistance as well as their migratory capacity and invasiveness [17,21]. Similar to this and to the pro-migratory effect of extracellular YB-1 in inflammation [22], secreted YB-1 enhances migration, invasion, and anchorage-independent growth of melanoma cells. The fact that YB-1 secretion increases with tumour progression further indicates a functional role of extracellular YB-1 in the metastatic process of melanoma cells.

Initial evidence suggesting a potential auto-stimulation of melanoma cell metastasis was provided by both experimental data involving pro-invasive exosomes [55] as well as by a proteomic profiling of secretomes produced by metastatic melanoma cell lines, which identified several candidate proteins that support the migration and invasion of tumour cells [16]. Indeed, secreted factors are powerful mediators, as next to autocrine effects, they allow propagation of functional effects by paracrine stimulation of neighbouring cells. By these means, therapy-induced secretomes of MAPK pathway inhibitor resistant melanoma cells have been shown to not only stimulate their outgrowth and dissemination, but also support the survival of drug-sensitive adjacent cancer cells [8]. Using a similar mechanism, single melanoma cells with enhanced YB-1 secretion could therefore profoundly enhance the metastatic capacity of several melanoma cells within the primary tumour. Indeed, we can show that YB-1 supplementation to melanoma cells with reduced YB-1 secretion due to gene silencing or knockout raises the migratory activity of these cells to the level of the corresponding YB-1 proficient cells with a consistent genetic background but robust intrinsic YB-1 secretion.

In conclusion, we can substantially extend the emerging role of YB-1 as an extracellular mediator with this study and further strengthen its importance as a powerful oncogenic player in melanoma cell biology.

## 5. Conclusions

In summary, YB-1 can be actively released from melanoma cells via unconventional protein secretion. The amount of secreted YB-1 strongly increases in melanoma cell lines originating from invasively growing and metastatic tumours. Simultaneously, we show that extracellular YB-1 promotes melanoma cell migration, invasion, and anchorage-independent growth. As these are important steps during the metastatic process, YB-1 secretion might not only serve as a novel marker for tumour aggressiveness but also represent an important functional player in melanoma metastasis.

## Supplementary Materials

The following are available online at https://www.mdpi.com/2072-6694/12/8/2328/s1, Figure S1: YB-1 release from melanoma cells correlates with melanoma progression stage. Figure S2: Melanoma progression stage dependent YB-1 release occurs actively. Figure S3: YB-1 secretion does not depend on the classical secretory pathway. Figure S4: YB-1 secretion occurs via the non-classical secretory pathway. Figure S5: YB-1 secreted from melanoma cells occurs mainly as a free protein. Figure S6: YB-1 secreted from melanoma cells is not packed into extracellular vesicles. Figure S7: YB-1 levels can be effectively downregulated in melanoma cells. Figure S8: *YBX1* can be effectively knocked out in melanoma cells. Figure S9: Extracellular YB-1 stimulates the migratory capacity of melanoma cells. Figure S10: Manipulation of YB-1 levels in melanoma cells does not affect cell growth but extracellular YB-1 content. Figure S11: Extracellular YB-1 does not influence growth of melanoma cells irrespective of intracellular YB-1 expression levels. Figure S12: Specificity of the stimulatory effect on wound closure by recombinant YB-1.
