# Supplementary material for: Tumour Progression Stage-Dependent Secretion of YB-1 Stimulates Melanoma Cell Migration and Invasion
Source: Cancers (Basel). 2020 Aug 18;12(8):2328. doi: 10.3390/cancers12082328 (PMC7464723; doi:10.3390/cancers12082328)
Supplement: Supplementary file 1 [file cancers-12-02328-s001.pdf]

cancers

MDPI

Supplementary Materials

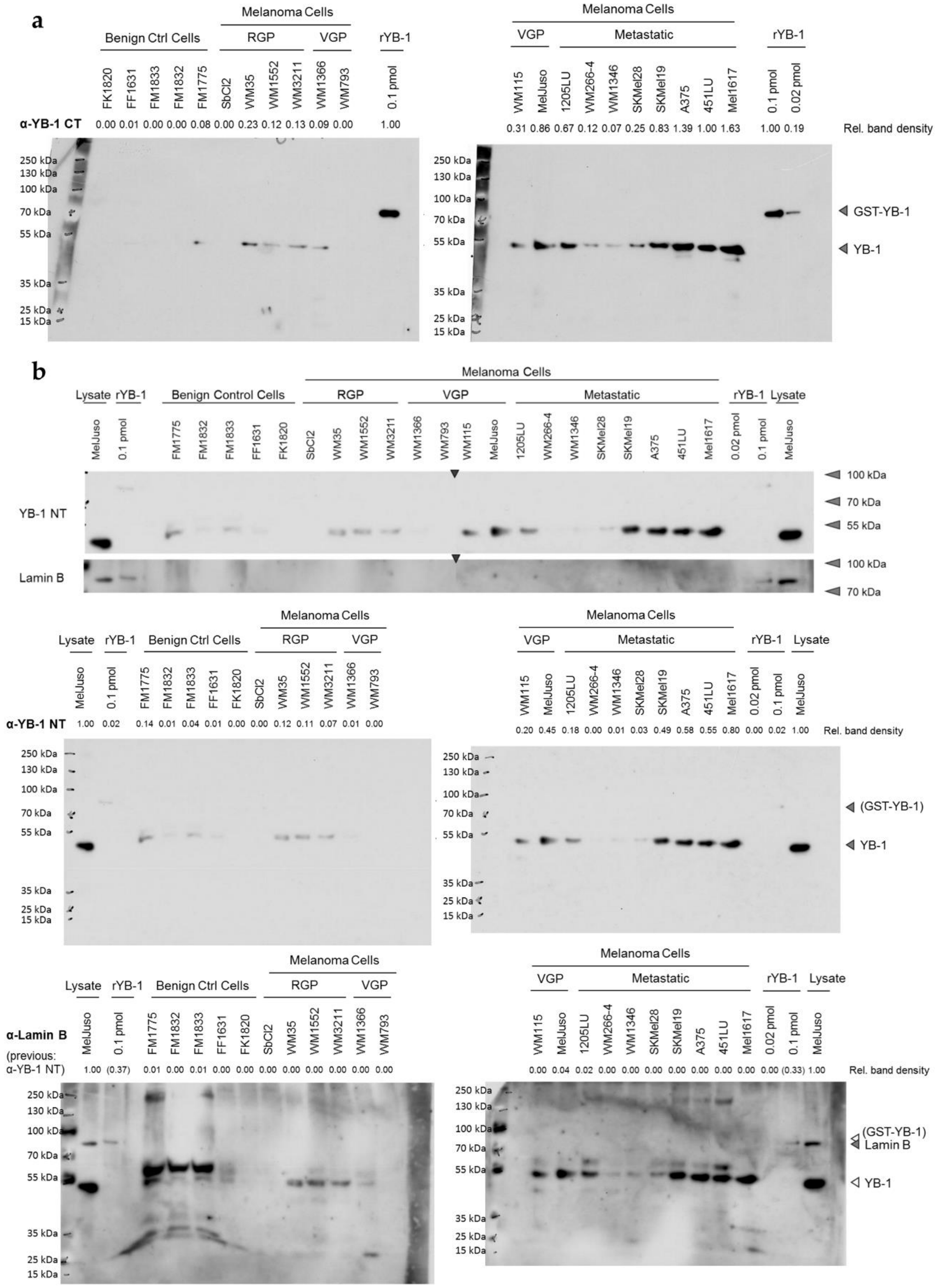

Figure S1. YB-1 release from melanoma cells correlates with melanoma progression stage. (**a**,**b**) Immunoblot analysis of 48 h-conditioned serum-free cell culture supernatants of benign control cells (melanocytes (FM), fibroblasts (FF), keratinocytes (FK)) and of melanoma cell lines derived from metastatic melanomas or primary melanomas in the radial (RGP) or vertical growth phase (VGP)

using a YB-1 specific antibody directed against its C-terminus (CT, **a**) or its N-terminus (NT, **b**). Whole (**a**,**b**) and cropped (**b**) immunoblots are shown. The boundary between cropped gels is marked by an arrowhead (**b**). Lamin B serves as a marker for unspecific protein release due to cell death (**b**). Cell lysates were used as positive control for Lamin B detection and together with recombinant YB-1 (rYB-1) as a protein standard allowing inter-gel comparisons (**a**,**b**). Due to its N-terminal GST-tag, rYB-1 detection by α-YB-1 NT is impaired. Densitometric analysis of the presented bands was conducted and relative values normalized to the respective protein standard are indicated.

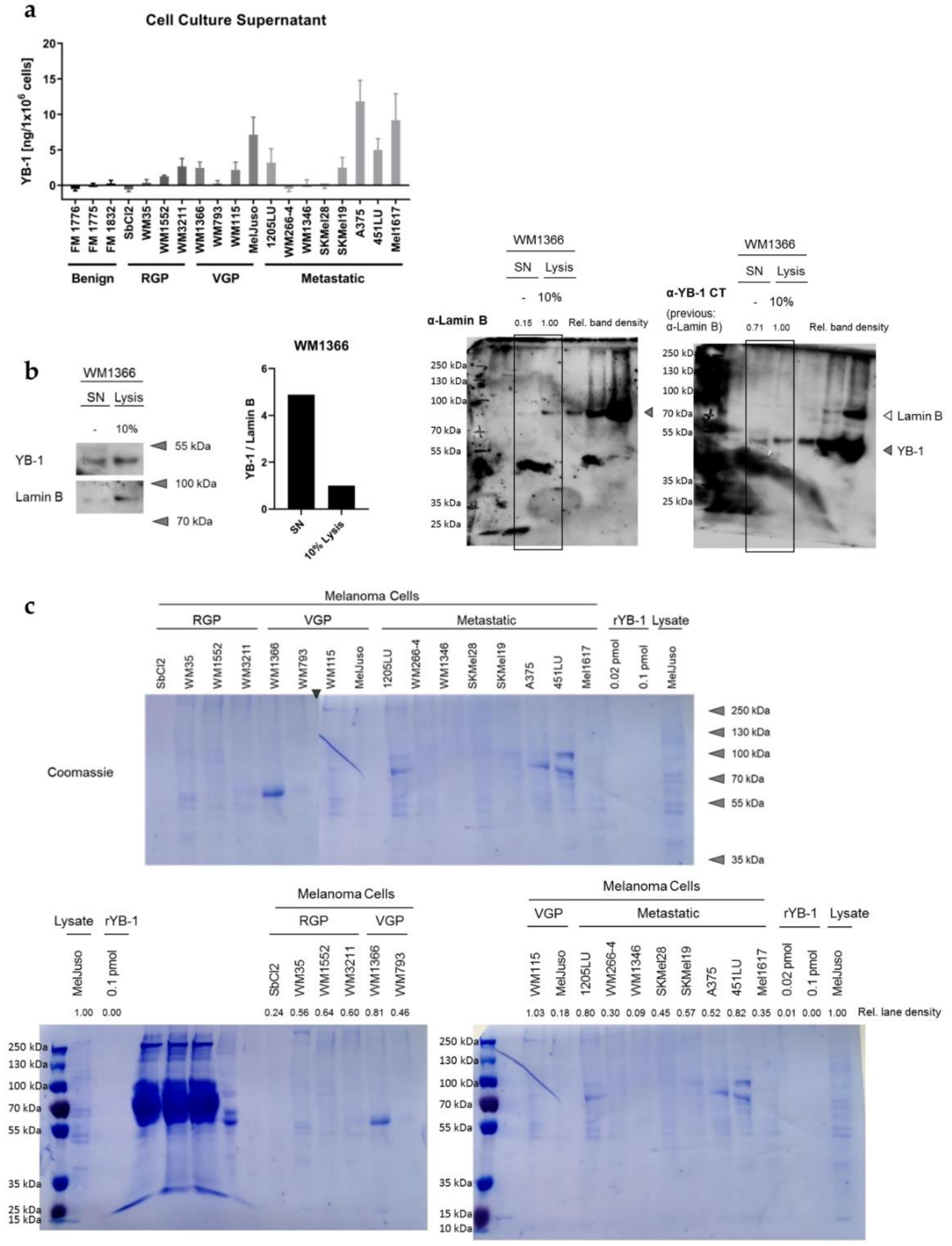

Figure S2. Melanoma progression stage dependent YB-1 release occurs actively. (**a**) YB-1 specific ELISA of melanoma cell- or melanocyte-conditioned cell culture supernatants (N = 3, mean ± standard deviation (SD) of each cell line). (**b**) Comparative Western Blot analysis of 48 h-conditioned cell culture supernatant and unconditioned medium containing WM1366 cell lysate equivalent to 10% cell death. Semi-quantification of YB-1 content was performed and normalized to the intracellular marker Lamin B. Relative band densities are indicated above the uncropped immunoblots. (**c**) Total protein in the conditioned culture supernatants was visualized by Coomassie staining of the membranes. The

boundary between the two in parallel processed gels is marked by an arrowhead and uncropped images shown below including relative signal intensities assessed by densitometric analysis of the individual lanes.

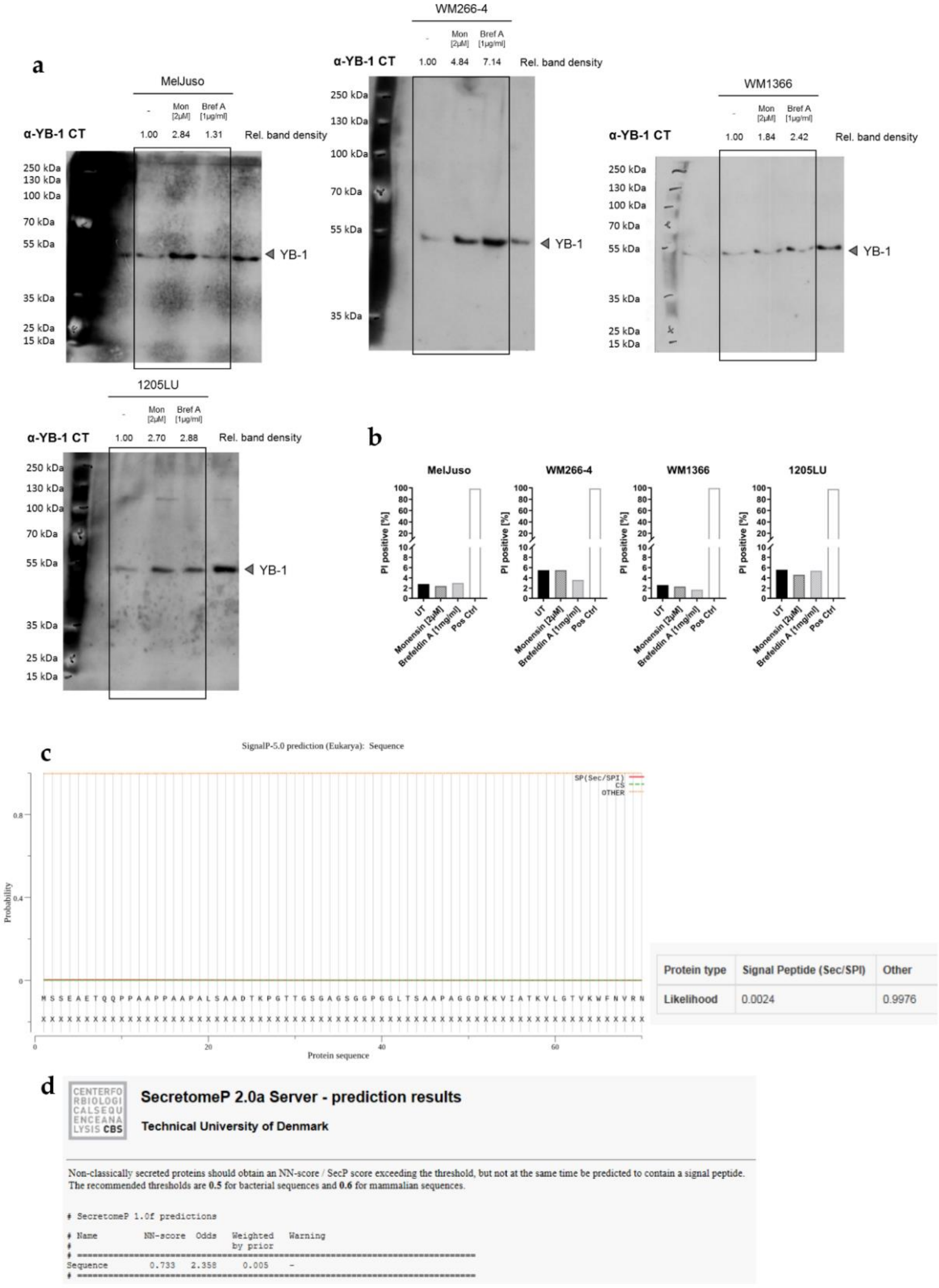

Figure S3. YB-1 secretion does not depend on the classical secretory pathway. (**a**) Western Blot analysis of serum-free cell culture supernatants after inhibition of classical protein secretion by monensin or brefeldin A. Uncropped immunoblots corresponding to Figure 2a and relative band densities are shown. (**b**) Relative viability of the melanoma cells used for supernatant collection after treatment with monensin or brefeldin A as assessed by flow cytometric detection of propidium iodide uptake. Cells treated with 70% EtOH served as positive controls with a compromised membrane integrity. (**c**) Prediction of signal peptide occurrence within the YB-1 protein sequence conducted with

the SignalP-5.0 webtool provided by Almagro Armenteros, et al. [1]. (**d**) Feature-based prediction of non-classical protein secretion for YB-1 using the SecretomeP 2.0 webtool described in Bendtsen, et al. [2].

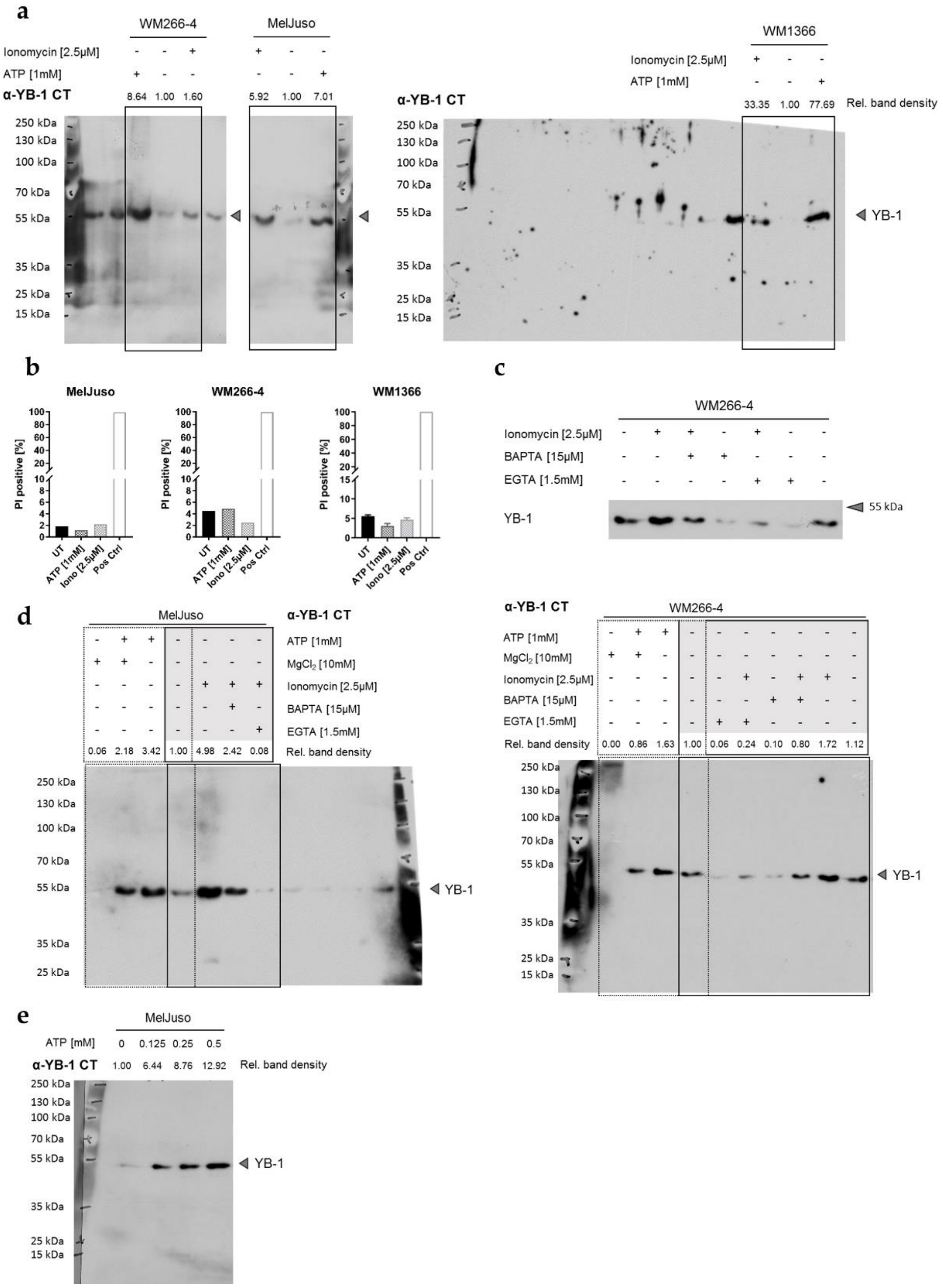

Figure S4. YB-1 secretion occurs via the non-classical secretory pathway. (**a**) Western Blot analysis of serum-free culture supernatants after stimulation of the melanoma cells with the Ca^{2+} ionophore ionomycin and ATP. Uncropped immunoblots corresponding to Figure 2b and relative band densities are shown. (**b**) Relative viability of the melanoma cells used for supernatant collection after treatment with ATP or ionomycin as assessed by flow cytometric detection of propidium iodide uptake. Cells treated with 70% EtOH served as positive controls with a compromised membrane integrity. (**c**–**e**) Western Blot analysis of serum-free culture supernatants after stimulation of the melanoma cells with the combination of ionomycin and the calcium chelators BAPTA or EGTA (**c**, cropped; **d**, whole

immunoblots corresponding to Figure 2c and Figure S4c), with the combination of ATP and the ATP complexing agent $MgCl_2$ (**d**, whole immunoblots corresponding to Figure 2e) or with increasing ATP concentrations (**e**, whole immunoblots corresponding to Figure 2d). Relative band densities are indicated.

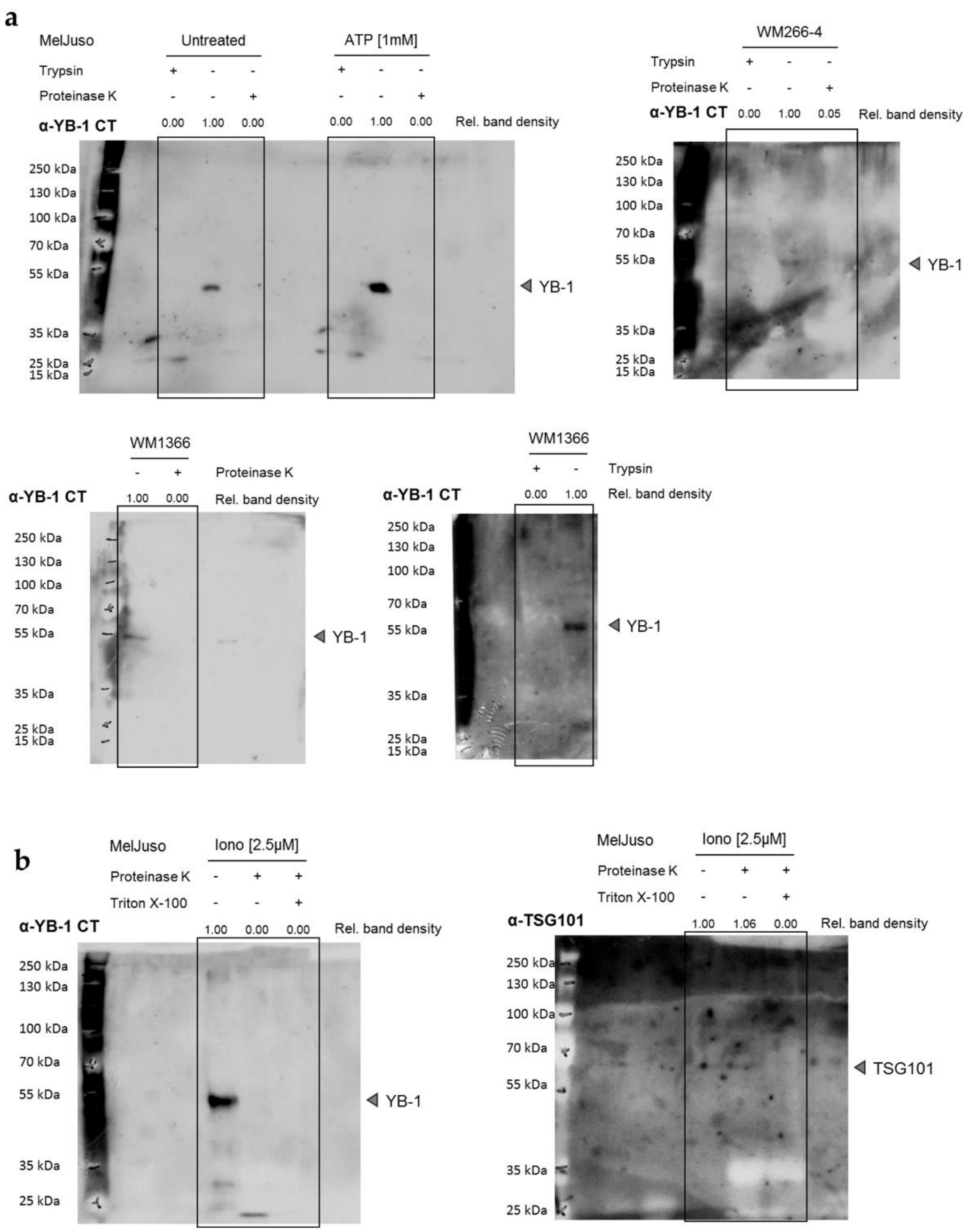

Figure S5. YB-1 secreted from melanoma cells occurs mainly as a free protein. (**a**,**b**) Protease protection assay followed by Western Blot analysis for YB-1 in serum-free cell culture supernatants. Uncropped immunoblots corresponding to Figure 3a,b (**a**) and Figure 3c (**b**) as well as relative band intensities are shown.

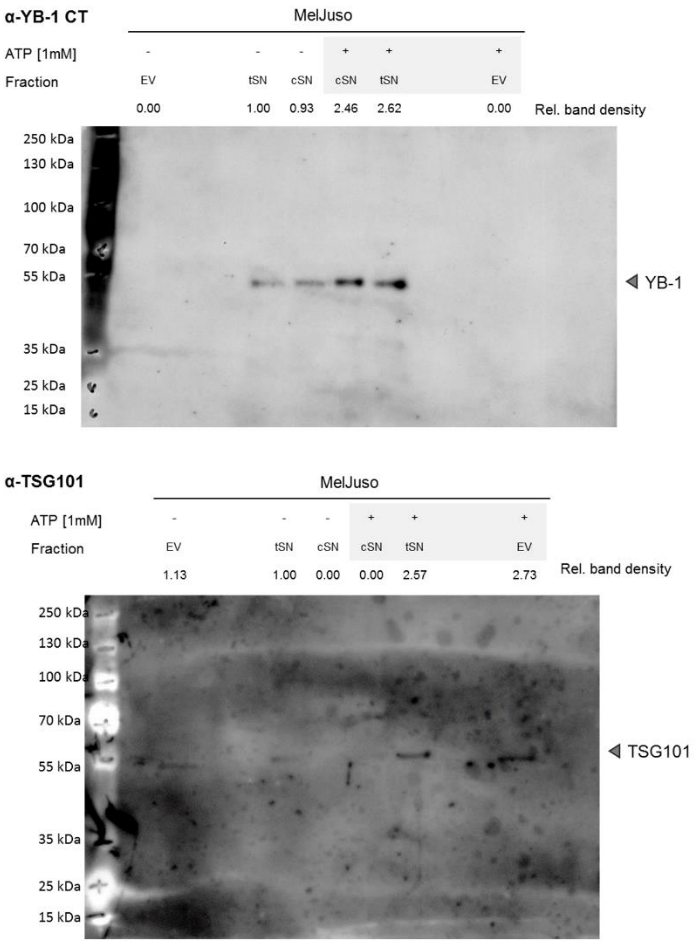

Figure S6. YB-1 secreted from melanoma cells is not packed into extracellular vesicles. Western Blot analysis of MelJuso-conditioned serum-free cell culture supernatants before (total SN, tSN) and after (cleared SN, cSN) purification of extracellular vesicles (EV) by ultracentrifugation. Uncropped immunoblots corresponding to Figure 3d and relative band intensities are shown.

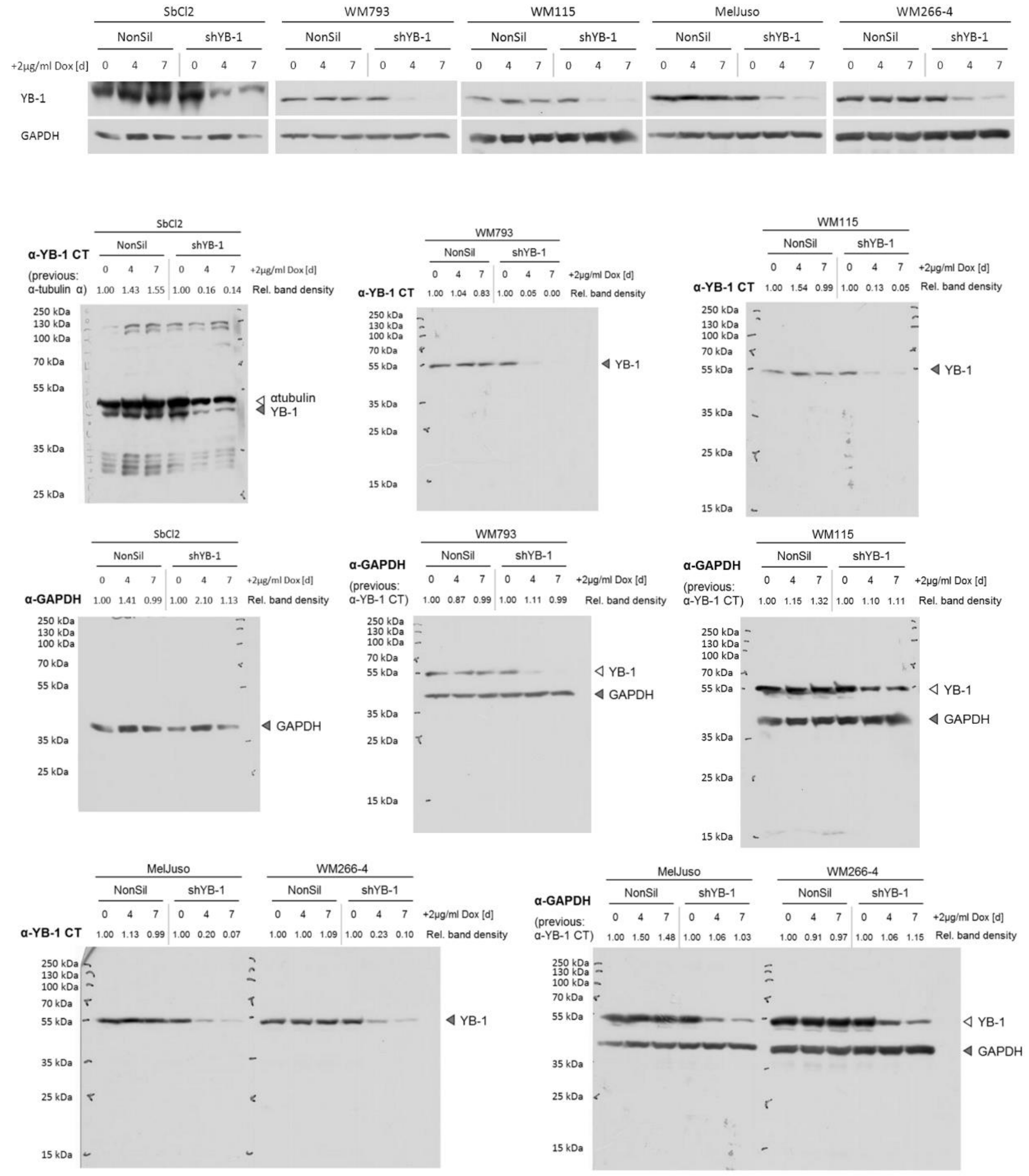

Figure S7. YB-1 levels can be effectively downregulated in melanoma cells. Western Blot analyses of YB-1 protein expression using whole cell lysates from melanoma cells harbouring an inducible YB-1 specific shRNA (shYB-1) or control shRNA (NonSil). Protein expression was assessed at the indicated timepoints after induction with 2 µg/mL doxycycline. GAPDH was detected as a loading control. Cropped, the corresponding whole immunoblots and the relative band intensities are shown.

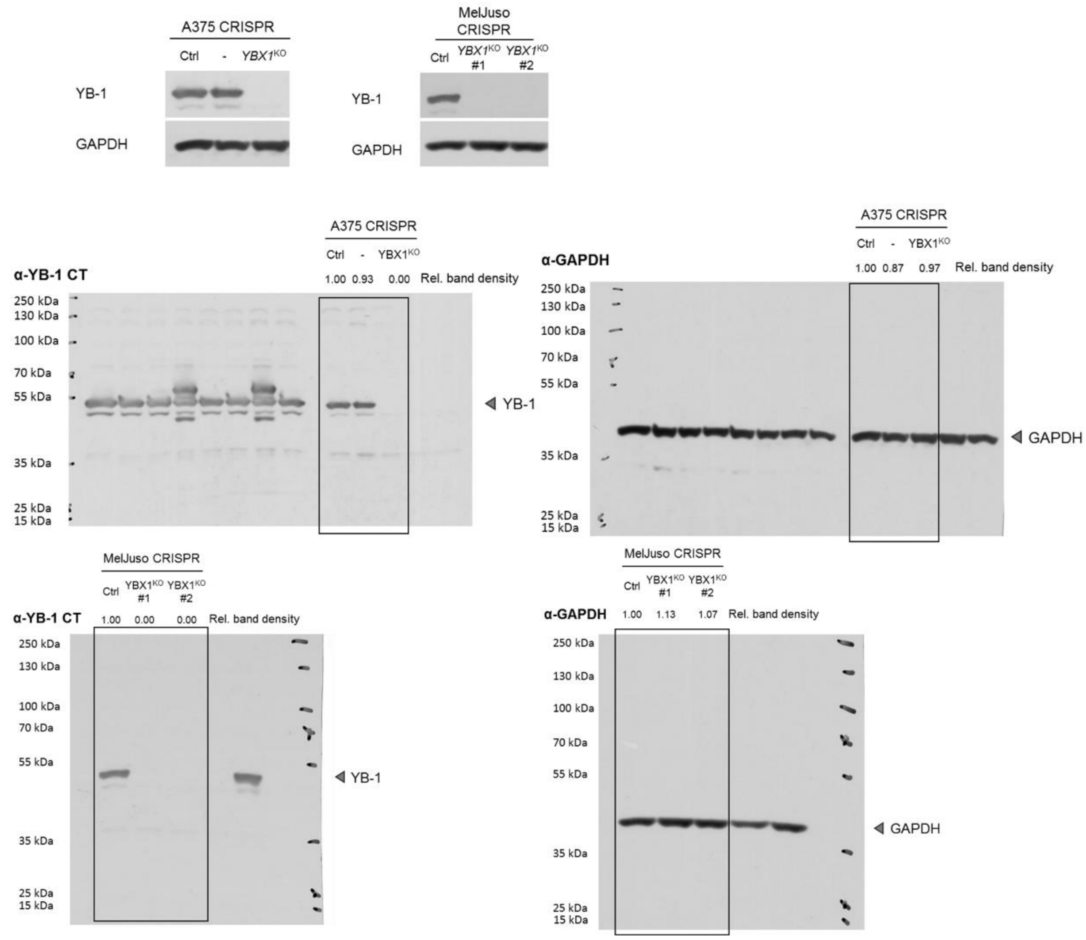

Figure S8. *YBX1* can be effectively knocked out in melanoma cells. Western Blot analyses of YB-1 protein expression using whole cell lysates from melanoma cells with CRISPR/Cas9 mediated *YBX1* knockout (CRISPR *YBX1*^{KO}) and the respective control cells (Ctrl). GAPDH was detected as a loading control. Cropped, the corresponding whole immunoblots and the relative band intensities are shown.

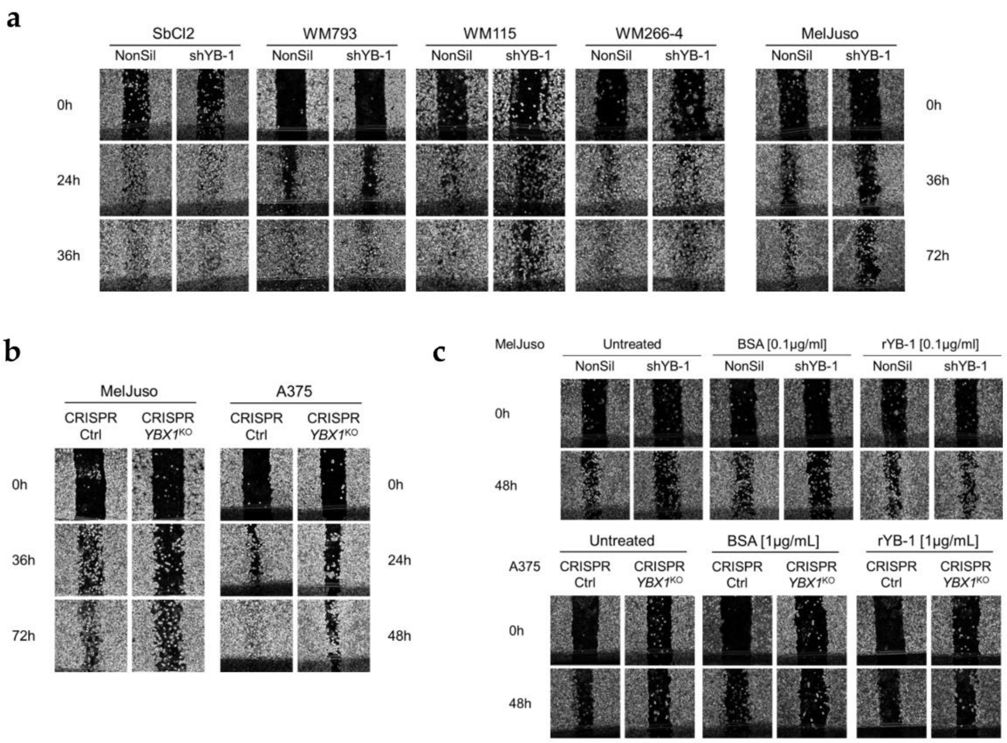

Figure S9. Extracellular YB-1 stimulates the migratory capacity of melanoma cells. (**a**,**b**) Representative pictures depicting wound closure over time by different melanoma cell lines with downregulated YB-1 expression (shYB-1-based RNA interference (**a**), CRISPR/Cas9 mediated *YBX1* knockout (**b**)) compared to the respective control cells. (**c**) Representative pictures of wound closure by melanoma cells with downregulated YB-1 expression (shYB-1 (top), *YBX1*^{KO} (bottom)) and the respective control cells after stimulation with recombinant YB-1 (0.1 µg/mL or 1 µg/mL) or BSA (0.1 µg/mL or 1 µg/mL).

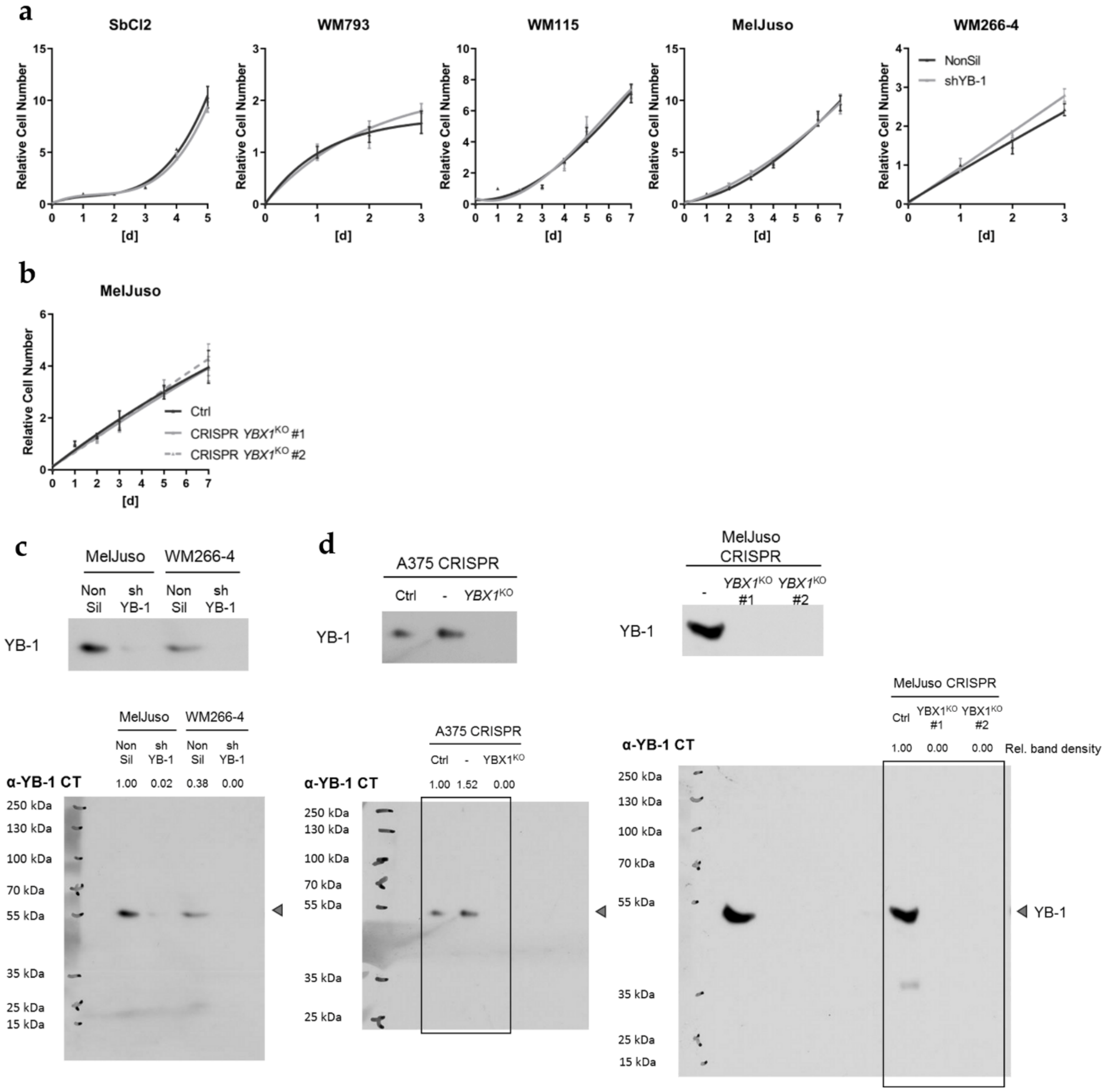

Figure S10. Manipulation of YB-1 levels in melanoma cells does not affect cell growth but extracellular YB-1 content. (**a**,**b**) Analysis of cell growth after downregulation of endogenous YB-1 levels using inducible YB-1 specific shRNA (shYB-1) (a) or CRISPR/Cas9-based *YBX1* knockout (CRISPR *YBX1*^{KO}) (b) in comparison to the respective control cells. In case of the inducible shRNA system, cells were pre-induced for 3 d and continuously treated with 2 µg/mL doxycycline. Growth curves were generated using a cell viability assay (MUH assay). Signals were normalized to the respective cell viability at day 1 (mean ± SD, *n* = 5 (a) or *n* = 8 (b)). (**c**,**d**) YB-1 specific immunoblot analysis of serum-free cell culture supernatant derived from melanoma cells with knockdown of endogenous YB-1 levels (shYB-1, c) or *YBX1* knockout (*YBX1*^{KO}, d) as well as the respective control cells. Cropped, the corresponding whole immunoblots and the relative band intensities are shown.

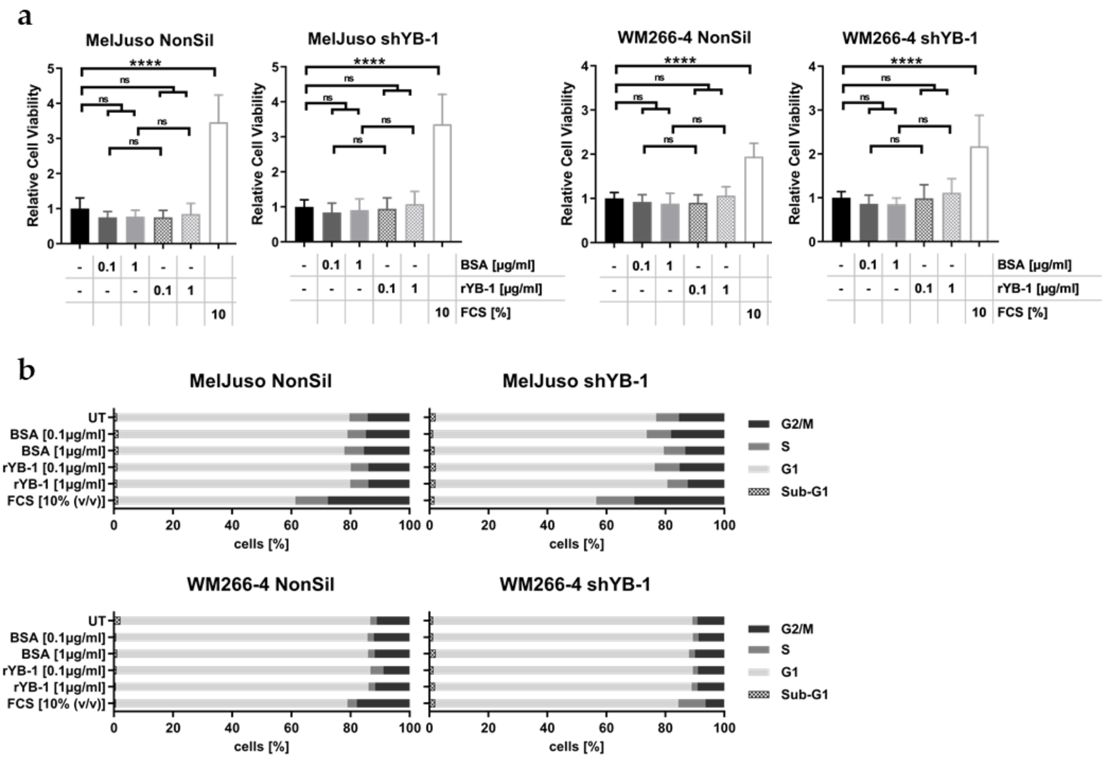

Figure S11. Extracellular YB-1 does not influence growth of melanoma cells irrespective of intracellular YB-1 expression levels. (**a**) Relative number of viable melanoma cells expressing YB-1 specific shRNA (shYB-1) or control shRNA (NonSil) upon treatment with recombinant YB-1 (0.1 µg/mL, 1 µg/mL), BSA (0.1 µg/mL, 1 µg/mL) or 10% FCS over 2 d. Signals were normalized to the respective untreated controls (mean ± SD, N = 3 (MelJuso) or N = 2 (WM266-4) with *n* = 5). Significance was determined with one-way ANOVA and subsequent Tukey's multiple comparison test. (**b**) Flow cytometric cell cycle analysis of shYB-1 and control shRNA (NonSil) expressing melanoma cells stimulated with recombinant YB-1 (0.1 µg/mL, 1 µg/mL), BSA (0.1 µg/mL, 1 µg/mL) or 10% FCS for 24 h. Fractions of cells in sub-G1, G1, S and G2/M phase were quantified (mean ± SD, representative experiment of N = 2 with *n* = 3).

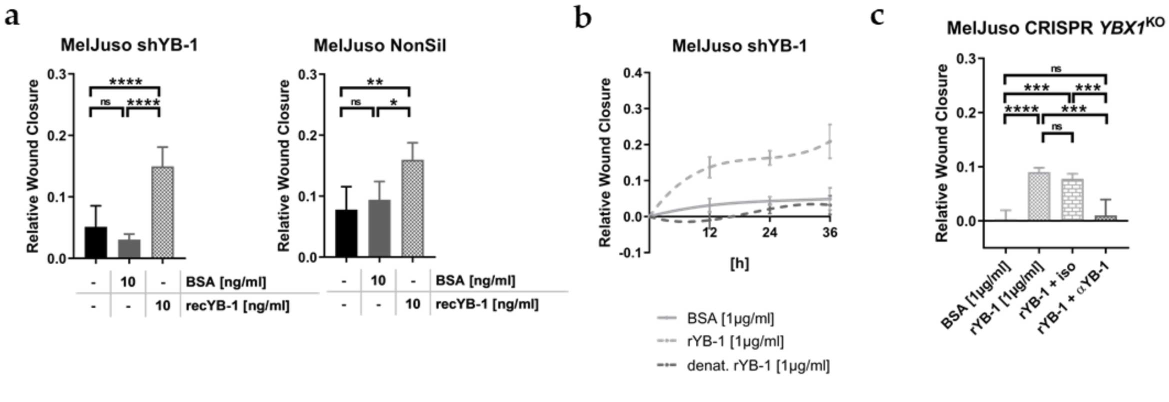

Figure S12. Specificity of the stimulatory effect on wound closure by recombinant YB-1. (**a**–**c**) Wound closure by MelJuso melanoma cells with downregulated YB-1 expression (shYB-1-based RNA interference (**a**,**b**), CRISPR/Cas9 mediated *YBX1* knockout (c)) after stimulation with low concentrations of recombinant YB-1 (10 ng/mL) or BSA (10 ng/mL) (**a**; mean ± SD; *n* = 4), with recombinant YB-1 in its native form or after heat denaturation (**b**, mean ± SD; *n* = 4), with recombinant YB-1 in combination with a YB-1 specific blocking antibody or its isotype control (**c**, mean ± SD; N = 2 with *n* = 8). Significance was determined with one-way ANOVA and subsequent Tukey's multiple comparison test where indicated.

References

1. Almagro Armenteros, J.J.; Tsirigos, K.D.; Sonderby, C.K.; Petersen, T.N.; Winther, O.; Brunak, S.; von Heijne, G.; Nielsen, H. SignalP 5.0 improves signal peptide predictions using deep neural networks. *Nat. Biotechnol.* **2019**, *37*, 420–423, doi:10.1038/s41587-019-0036-z.
2. Bendtsen, J.D.; Jensen, L.J.; Blom, N.; Von Heijne, G.; Brunak, S. Feature-based prediction of non-classical and leaderless protein secretion. *Protein Eng. Des. Sel.* **2004**, *17*, 349–356, doi:10.1093/protein/gzh037.

© 2020 by the authors. Licensee MDPI, Basel, Switzerland. This article is an open access article distributed under the terms and conditions of the Creative Commons Attribution (CC BY) license (http://creativecommons.org/licenses/by/4.0/).
